# Bichloride of Mercury as a Germicide

**Published:** 1887-12

**Authors:** H. E. Stroud

**Affiliations:** Grand Junction, Colorado


### BICHLORIDE OF MERCURY AS A GERMICIDE.

H. E. STROUD, M. D., GRAND JUNCTION, COLORADO.

When Paracelsus, in 1600, used this antiseptic, upon the theory of preventing or destroying germs, and thereby checking sepsis, which was of such frequent occurrence, before the advent of germacides, he was regarded by the profession as a crank; and such were ancient prejudices that the use of this king of germacides died with its originator, to be revived by Devaine in 1873, and Bilbroth, in 1874, and now it stands permanently the chief of antiseptics. Much has been written upon the dangers attending its reckless use in large, open wounds, and in cavities where free drainage cannot be secured; and especially are its dangers in gynecological and obstetrical practice magnified. Much of the warning is opportune, but with the same therapeutic knowledge and caution that govern the use of other powerful remedies, this has a place in our armamentarium.

Bichloride of mercury is a violent poison, causing intestinal pain, diarrhœa, etc., and no matter in what way introduced, the constitutional symptoms are the same, and in using it the idea is to use a sufficiently strong solution to destroy the germs, but not strong enongh to work an injurious effect upon the person; but to decide what this is, requires consideration. As a rule, 1 to 500 is the strongest, but 1 to 1,000 or 2 000 is generally strong enough to fulfill the required mission. But in using the solution in a cavity where some of the fluid will be retained and absorbed, 1 to 4 or 5,000 should be used. In the case of puerperal septicæmia where time is everything, a strong solution may and often should be used, and the parts thoroughly irrigated afterwards with a very weak solution or clean water. In a wound where the microbes are active and the vital power low, we would use the strong solution and thoroughly wash with the weak solution; but, in ordinary cases a solution 1–2,000 will answer, and in my hands this plan has always succeeded. Nor have I had any constitutional trouble after using the stronger solution; but I am thoroughly alive to its dangers if not properly arranged.

A case came under my care as railroad surgeon. Mr. ——, was badly scalded in a railroad accident. From the middle of the thighs to the ankles were deeply burned. I saw him twelve hours after the accident, and dressed them with carron oil, and in a few days poultices were applied to relieve the throbbing pain, and openings were made to secure drainage, and cistern water used as a spray and wash ; also constitutional treatment, as required. The case progressed well for about three weeks, when symptoms of septic poisoning suddenly developed, the raw surfaces took on a grayish, ragged appearance ; temperature 104° ; tongue dark and dry. I used a solution 1-1,000 bichloride, and gave antipyrin. The next morning there was a great improvement ; the bichloride was continued, the fever subsided, when it was withdrawn. But the fever commenced again, and was promptly reduced. As this was a long case, I tried several times to use other germacides, but had to fall back on the bichloride. As they were such large surfaces, and I had read so much of the dangers and deaths following its use, I used after the first dressing 1–2,000, and never did wounds heal nicer. As the weather was getting hot here, I sent the patient to the Company Hospital, which is located in a higher and cooler altitude, where the case was completed.

My next case was one of septic fever and inflammation, resulting from retained placenta, following abortion. I cureted the uterus with a wire curet, and washed the organ out with a 1–1,000 solution ; afterwards with Dist Hamamelis and water, 1–20. The case did well ; and later an obstetrical case where I had to perform craniotomy, the vagina was washed with 1–2000, followed by Hamamelis and water, and no case did better, and as it was during our hottest weather, and there was some maternal injury, I credit the bichloride with being a prophylactic.

It is the only thing that will sterilize the hands, as they should be in obstetric practice, 1–500. In a comparative test, I see it ranks first as an injection in acute gonorrhœa ; also in ophthalmia neonatorum, as a local application 1–5,000 or 1–10,000. To those who have never used it the following rules may be of service: 1. Never use too strong a solution—rarely exceed 1–1,000. 2. Mix the solution in a non-metallic vessel. 3. The action is instantaneous ; so wash the surface off immediately with clean water, and thoroughly, and don't use it too often.

I cannot end this paper better than by recommending a little book worthy of any one's time to read, namely : "How We Treat Wounds To-Day," by R. T. Morris, of New York.

